# Secretome Analysis of the Banana Fusarium Wilt Fungi *Foc R1* and *Foc TR4* Reveals a New Effector OASTL Required for Full Pathogenicity of *Foc TR4* in Banana

**DOI:** 10.3390/biom10101430

**Published:** 2020-10-09

**Authors:** Dan Wang, Cunzhi Peng, Xingmei Zheng, Lili Chang, Bingqiang Xu, Zheng Tong

**Affiliations:** 1 Institute of Tropical Bioscience and Biotechnology, Chinese Academy of Tropical Agricultural Sciences, Haikou 571101, China; wangdan@itbb.org.cn (D.W.); pengcunzhi@itbb.org.cn (C.P.); konghua@itbb.org.cn (X.Z.); changlili@itbb.org.cn (L.C.); 2 Hainan Key Laboratory for Protection and Utilization of Tropical Bioresources, Hainan Institute for Tropical Agricultural Resources, Chinese Academy of Tropical Agricultural Sciences, Haikou 571101, China; 3 Haikou Experimental Station (Institute of Tropical Fruit Tree Research) Chinese Academy of Tropical Agricultural Sciences, Haikou 571101, China; 4 Key Laboratory of Banana Genetics and Improvement, Haikou 571101, China

**Keywords:** banana Fusarium wilt, *Fusarium oxysporum* f. sp. *cubense*, secretome, secretory protein, pathogenicity, O-acetylhomoserine (thiol)-lyase

## Abstract

Banana Fusarium wilt (BFW), which is one of the most important banana diseases worldwide, is mainly caused by *Fusarium oxysporum* f. sp. *cubense* tropic race 4 (*Foc TR4*). In this study, we conducted secretome analysis of *Foc R1* and *Foc TR4* and discovered a total of 120 and 109 secretory proteins (SPs) from *Foc R1* cultured alone or with banana roots, respectively, and 129 and 105 SPs respectively from *Foc TR4* cultured under the same conditions. *Foc R1* and *Foc TR4* shared numerous SPs associated with hydrolase activity, oxidoreductase activity, and transferase activity. Furthermore, in culture with banana roots, *Foc R1* and *Foc TR4* secreted many novel SPs, of which approximately 90% (*Foc R1*; 57/66; *Foc TR4*; 50/55) were unconventional SPs without signal peptides. Comparative analysis of SPs in *Foc R1* and *Foc TR4* revealed that *Foc TR4* not only generated more specific SPs but also had a higher proportion of SPs involved in various metabolic pathways, such as phenylalanine metabolism and cysteine and methionine metabolism. The cysteine biosynthesis enzyme O-acetylhomoserine (thiol)-lyase (OASTL) was the most abundant root inducible *Foc TR4*-specific SP. In addition, knockout of the *OASTL* gene did not affect growth of *Foc TR4*; but resulted in the loss of pathogenicity in banana ‘Brazil’. We speculated that OASTL functions in banana by interfering with the biosynthesis of cysteine, which is the precursor of an enormous number of sulfur-containing defense compounds. Overall, our studies provide a basic understanding of the SPs in *Foc R1* and *Foc TR4*; including a novel effector in *Foc TR4*.

## 1. Introduction

During the last century, the banana crop industry has experienced dramatic losses due to an epidemic of banana Fusarium wilt (BFW), caused by *Fusarium oxysporum* f. sp. *cubense* race 1 (*Foc R1*), which has more recently been intensified by *Foc* tropical race 4 (*Foc TR4*) in Cavendish banana [1]. However, the mechanism underlying BFW resistance in Cavendish banana at the genetic level is unclear, and biological control remains the mainstream to method of control [2].

Pathogens generate secretory proteins (SPs) that are critical for infection of the host and function in processes including degradation of the cell wall and biomacromolecules as well as contributing to modulation of metabolic processes and immune responses [3,4]. Studies of SPs have revealed many pathogenic mechanisms. For example, AvrE-family Type-III effector proteins (T3Es) contribute to the virulence of multiple plant-pathogenic species by disturbing pathogen-associated molecular pattern-triggered immunity (PTI) via direct interaction with the B’ regulatory subunits of phosphatase 2A (PP2A) heterotrimeric enzyme complexes [5]. A cysteine-rich small SP SsSSVP1 in the necrotrophic phytopathogen *Sclerotinia sclerotiorum* was demonstrated to interact with QCR8, a subunit of the cytochrome b-c1 complex in the mitochondrial respiratory chain in plants, and also to induce plant cell death [6]. Avr3a-like effectors from *Phytophthora* spp. were shown to target the plant cinnamyl alcohol dehydrogenase 7 (CAD7) subfamily members to enhance the suppression of PTI functions, including callose deposition, reactive oxygen species burst, and WRKY33 expression [7]. A ‘WY-domain’ RXLR effector PiSFI3 of the potato blight agent *Phytophthora infestans* was found to suppress PTI via interaction with the U-box-kinase protein StUBK, a positive regulator of specific PTI pathways [8]. Disease resistance proteins were also discovered through their effectors. The secreted fungal chorismate mutase, Cmu1, is thought to affect biosynthesis of the plant immune signal salicylic acid by channeling chorismate into the phenylpropanoid pathway, and a maize-encoded kiwellin, ZmKWL1, specifically targets and blocks the catalytic activity of Cmu1 [9].

Secretome profiling and prediction have been used to identify SPs of many pathogens, including *Tilletia horrida* [10], *Fusarium proliferatum* [11], *Tilletia indica* [12,13], rose black spot [4], *Trichoderma virens* [14], *Arbuscular mycorrhizal* [15], and *Puccinia striiformis f. sp. tritici* [16]. In the banana fruit pathogen, *F. proliferatum*, 105 secretory proteins were predicted based on the pathogen proteome [11]. In *Foc TR4*, 2235 SPs were identified in one study, although the specific data were not shown [17]. Recently, some effectors have been shown to be necessary for full toxicity of *Foc TR4* in banana, including Six1 [18], Six8 [19], and CP1 [20]. However, a more comprehensive understanding of the pathogenesis of *Foc R1* and *Foc TR4* is required to identify reliable countermeasures for the control of BFW. In this study, we identified many SPs generated in *Foc R1* and *Foc TR4* in culture alone or during the early stages of infection of banana, and identified a root inducible *Foc TR4*-specific OASTL, which is indispensable for *Foc TR4* pathogenicity in banana.

## 2. Materials and Methods

### 2.1. Plant Materials and Pathogen Infection

Banana ‘Brazil’ plants (*Musa acuminate L*., genome group AAA) were obtained from the Banana Tissue Culture Center (Institute of Banana and Plantains, Chinese Academy of Tropical Agricultural Sciences, Danzhou, Hainan, China), which were vitro-propagated by sucker tissues of banana. Plants at the full fifth-leaf stage were grown at approximately 30 °C with an approximate 14/10-h light/dark photoperiod and used in inoculation experiments. *Foc R1* and *Foc TR4* strains were gifted from Banana Industry Technology Research Group, Institute of Tropical Bioscience and Biotechnology, Chinese Academy of Tropical Agricultural Sciences. Both *Foc R1* and *Foc TR4* were cultured at 28 °C with shaking at 200 rpm for 48 h in KK culture medium (0.1% K_2_HPO_4_, 0.05% KCl, 0.05% MgSO_4_·7H_2_O, 0.001% Fe-NaEDTA, 0.2% L-ASP, 1% D-galactose, *w*/*v*, pH 5.0). The mycelia were removed by filtration using sterilized lens paper, and the conidia in the filtrate were collected by centrifugation. The conidia were suspended to a concentration of 10^7^ cfu mL^−1^ using 1/2 Hoagland nutrient solution. Plant roots were soaked in 200 mL 1/2 Hoagland solution with conidia for 72 h in a light incubator at 28 °C with a 16/8-h light/dark photoperiod before the suspension was collected. The plants were then replanted in pots and cultured in a greenhouse at approximate 32 °C with an approximate 14/10-h light/dark photoperiod for further observation.

### 2.2. Secretory Protein Extraction

After banana roots were soaked in conidia suspension for 72 h, the SPs of *Foc R1* and *Foc TR4* were collected and extracted. The conidia suspension was centrifuged at 1.2 × 10^4^ rpm for 15 min at 4 °C, and the supernatant were filtered (0.45 μm pore size filter; Sangon Biotech, Shanghai, China). The SPs in the filtrate were extracted using the method as previously described [21]. In brief, an equal volume of protein extraction buffer (100 mM ethylenediaminetetraacetic acid, 100 mM Tris-HCl, 50 mM borax, 50 mM vitamin C, 30% sucrose, 2% β-mercaptoethanol, 1% Triton X-100, and 1% polyvinylpyrrolidone cross-linked, pH 8.0) was added to the filtrate and the mixtures were divided into six portions. An equal volume of Tris-saturated phenol (pH > 7.8) was added to one of the six portions. The mixtures were vortexed thoroughly for 5 min and centrifuged at 1.2 × 10^4^ rpm for 5 min at 4 °C. The phenol-based upper phase was then transferred into the next portion of the sample, and the extraction procedure was repeated until all portions had been extracted using the same phenol. The final upper phenol phase was transferred to a new centrifuge tube, and five volumes of ammonium sulfate-saturated methanol (AM) were added to precipitate proteins for at least 12 h at −20 °C. After centrifugation at 1.2 × 10^4^ rpm for 15 min at 4 °C, the SPs precipitated by AM were collected and resuspended in lysis buffer (7 M urea, 2 M thiourea, 4% CHAPS, 2% DTT, pH 8.5). Protein concentration was determined by Bradford assay using BSA as the standard [21].

### 2.3. Protein Identification by HPLC-ESI-MS/MS

Samples (100 μg) of SPs obtained from *Foc R1* and *Foc TR4* were reduced and alkylated by dithiothreitol and iodoacetamide, respectively, before trypsin digestion. The peptides were analyzed by HPLC-ESI-MS/MS (Orbitrap Fusion Lumos high resolution nanospray ESI-MS/MS mass spectrometer, Thermo Fisher Scientific, Waltham, MA, USA). The trap and elute mode was used to separate the samples with the EASY-nLC 1200 nano HPLC system (Thermo Fisher Scientific, Waltham, MA, USA) equipped with a trap column (Phenomenex, 2 cm, C18, 5 μm 125 Å) and a separation column (Phenomenex, 10 cm, C18, 3 μm, 125 Å). Each cycle consisted of TOF-MS spectrum acquisition for 3 s (mass range 300–2000 Da), followed by acquisition of MS peaks in up to 30 MS/MS spectra (3.2 s each scan, mass range 400–1200 Da). Mass spectrometer recalibration was performed at the start of the analysis of each sample using a β-galactosidase digest standard. Mass spectrometric data were transferred to Spectranaut software (Biognosys) and searched against a self-constructed database derived from the NCBI database (*Foc R1* database, BioProject: PRJNA174274, 15,441 component sequences; *Foc TR4* database, BioProject: PRJNA174275, 1.4459 × 10^4^ component sequences). The following sample parameters were used: trypsin digestion, number of missed cleavages set to 2, fixed and variable modifications set to carbamidomethylation (C) and oxidation (M), and mass tolerances for peptide and fragment ions set to 0.02 Da. An unused confidence score of 1.3 was used as a qualification criterion. Protein quantification was performed for identified proteins with at least two peptides matched with higher than 95% confidence and a false discovery rate (FDR) ≤ 1%.

### 2.4. Protein Bioinformatics Analysis

The identified proteins were searched against the AgBase database (Version 2.00, Arizona Board of Regents, Phoenix, AZ, USA, https://agbase.arizona.edu/cgi-bin/tools/GOanna.cgi) to confirm their functions. The input file of protein sequences was used to add gene ontology (GO) annotations to those predicted based on sequence homology in the AgBase-UniProt database analysis. The corresponding analysis parameters were as follows: number of target sequences = 5, Pct identity filter (%) = 70, query coverage filter (%) = 70. GO pathway analysis was performed using WeGO software (Version 2.0, The Beijing Genomics Institute, Shenzhen, China, http://wego.genomics.org.cn/) to access to information for cellular component, biological process, and molecular function. The KEGG website (https://www.kegg.jp/blastkoala/) and OmicShare tools (http://www.omicshare.com/tools) were used to determine the proteins involved in KEGG pathways and for enrichment analysis. We submitted our protein sequences in a fasta file to KEGG website, and obtained the KO number and pathway information of each protein, which were then organized to KEGG lists for further analysis. The target protein list files were entered into the Enrichment Analysis of Dynamic KEGG tools (https://www.omicshare.com/tools/Home/report/koenrich) with the following parameters: background gene table type = KO and species type = fungus. The results were analyzed dynamically by chart interaction. Signal peptide (SP), mitochondrial transit peptide (mTP), chloroplast transit peptide (cTP), or thylakoid luminal transit peptide (luTP) were predicted by TargetP-2.0 (DTU Health Tech, Lyngby, Denmark, http://www.cbs.dtu.dk/services/TargetP/) with the analysis parameters of Organism group = Non-Plant and Output format = Shot output.

### 2.5. RT-PCR Analysis

Total RNAs were extracted using the RNAprep Pure Plant Plus Kit (TIANGEN Biotech, Beijing, China), and cDNAs were synthesized using the RevertAid First Strand cDNA Synthesis Kit (Thermo Fisher Scientific, Waltham, MA, USA) according to the manufacturers’ instructions. Semi-quantitative RT-PCR analysis was performed with *Foc* universal 18S rRNA as the internal control. The primers used for RT-PCR analysis are listed in Supplementary Table S5. PCR reactions were performed in a total volume of 20 μL containing 1 μL cDNA as the template. The PCR conditions were as follows: initial denaturation for 5 min at 94 °C, followed by 30 cycles of denaturation for 20 s at 95 °C, annealing for 20 s at 58 °C, and elongation for 30 s at 72 °C, with a final incubation for 5 min at 72 °C after the last cycle. The PCR products (5 µL) were separated by 1.0% agarose gel electrophoresis and visualized using GoldView Nucleic Acid Stain (SBS Genetech, Beijing, China) and OI 100 fully automated gel imaging systems (BIO-OI, Guangzhou, China).

### 2.6. Fungal Vector Construction

The OASTL knockout vector was constructed using the pCT74HP-eGFP vector (Supplementary Figure S3). pCT74HP-eGFP was reconstructed from pCT74 [22] by replacing the *sGFP* gene with the multiple cloning site (*Hind* III-*EcoR* V-*Nhe* I-*Stu* I-*Bgl* II), removing an intron of the *ToxA* promoter (the original promoter contains the 5′ untranslated region of the *ToxA* gene, and the intron is in the region), and inserting eGFP into the vector at the *Nhe* I and *Stu* I sites. For the gene knockout vector construction, the left (633 bp) and right (729 bp) flanking sequences of *OASTL* coding region was inserted into pCT74HP-eGFP at the *Xba* I and *BamH* I sites and *Xho* I and *Kpn* I sites, respectively. Primers used in the constitution are shown in Supplementary Table S5.

### 2.7. Foc Transformation

To prepare the protoplast, fresh mycelia of *Foc TR4* were collected in three layers of filter paper, and washed three times with 20 mL 0.8 M NaCl. Mycelia (0.4 g) were digested in 8 mL lysate (10 mg mL^−1^ collagenase (Sigma, St. Louis, MO, USA) and 10 mg mL^−1^ lysozyme (Sigma, St. Louis, MO, USA), in 0.8 M NaCl) at 28 °C with shaking at 80 rpm for 3–4 h. The filtrates (containing protoplasts) were obtained by passing the suspension across five layers of sterilized lens paper. The filtrates were centrifuged at 5000 rpm for 10 min room temperature, and the precipitates (protoplasts) were washed once with 20 mL 0.8 M NaCl and twice with 5 mL STC (1.2 M sorbitol, 10 mM Tris-HCl pH 7.5, 50 mM CaCl_2_). Protoplasts were resuspended with STC to a final volume of 200 μL and transferred into 15 mL sterilized centrifuge tubes. In the process of protoplast transformation, 50 μL DNA solution containing 5–10 μg PCR products and 50 μL 60% PTC (60% PEG4000, 10 mM Tris-HCl pH 7.5, 50 mM CaCl_2_) were added sequentially to the protoplasts, mixed well and placed in an ice bath for 30 min. PTC (2 mL) was then added to the protoplasts in a dropwise manner, gently mixed and placed at room temperature for 20 min before adding 3 mL regeneration medium (200 g potato boiled liquid and 342 g sucrose per L) for static culture overnight at 28 °C in the presence of light. The culture was centrifuged at 5000 rpm for 10 min, and approximately 4 mL supernatant was discarded. The fungus precipitate was then suspended and transferred into regeneration agar medium (containing 0.9% agar) in the liquid state (40–50 °C) and mixed well before plating into the culture dishes. After solidification of the regeneration agar medium, a layer of resistance screening medium (potato dextrose agar (PDA) medium (200 g potato boiled liquid, 20 g sucrose, 0.9% agar per L), containing 200 μg mL^−1^ hygromycin B) was laid on it. Traces of transformants were obtained after 3–5 days of inverted culture at 28 °C. Individual conidia were selected from the transformants, and transferred to new PDA plates for continued culture four times. Then DNA and RNA of each clone were extracted for confirmation of the transformation or knockout events by PCR.

### 2.8. Pathogenicity Assay

Conidia suspensions (10^7^ cfu mL^−1^) were inoculated onto the roots of banana plants at the six-leaf stage using the immersion method as previously described. After immersion for 24 h, the bananas were planted into pots containing 1:1 nutrient soil and vermiculite and cultured in a greenhouse at approximate 32 °C with an approximate 14/10-h light/dark photoperiod. *Foc4G* (*Foc TR4* transformant of empty vector control) was used as a positive control for inoculation. Each *Foc TR4* transformant was used to inoculate 12 banana plant roots as one biological replicate. The longitudinal sections of banana pseudo-stems were observed 3 days after inoculation. Whole plant phenotypes were observed 30 days after inoculation and the survival rates were calculated. Similar results were obtained in three independent experiments.

## 3. Results

### 3.1. Foc R1 and Foc TR4 Infection of Banana

**Figure 1:**
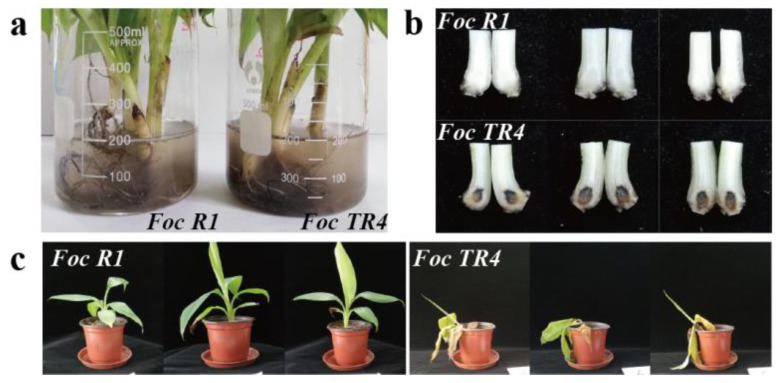
The observation of banana plants inoculated with *Foc R1* or *Foc TR4*. (**a**) Soaking the roots of banana plants in Horgland nutrient solution containing conidia of *Foc R1* or *Foc TR4* for 3 days; (**b**) the symptoms of the pseudo-stem sections of banana plants inoculated with *Foc R1* or *Foc TR4* after soaking for 3 days; (**c**) the statuses of the whole banana plants inoculated with *Foc R1* or *Foc TR4* after planting for another 11 days.

‘Brazil’ is a Cavendish banana, hence resistant to *Foc R1* but not *Foc TR4*. In the study, we investigated the pathogenic effects of *Foc R1* and *Foc TR4* in banana ‘Brazil’ plants by immersion of the roots in a fungal conidia suspension (Figure 1a). After immersion for 72 h, *Foc TR4* caused typical symptoms of BFW, with blackening of banana corms, while *Foc R1* did not cause such symptoms (Figure 1b). After immersion for 72 h, these banana plants were planted into soil, and after another 11 days, most of the plants soaked in *Foc TR4* conidia suspension showed yellowing and wilting of leaves, while plants soaked in *Foc R1* conidia solution remained healthy (Figure 1c). These results suggested that only *Foc TR4* caused BFW in banana ‘Brazil’.

### 3.2. Identification of Foc R1 and Foc TR4 SPs

We obtained the SPs of *Foc R1* and *Foc TR4* cultured alone or with banana roots in two independent experiments (Supplementary Table S1). In culture alone, 120 SPs were identified from *Foc R1* and 129 SPs were identified from *Foc TR4* (Supplementary Table S1). In culture with banana roots, 109 SPs were identified from *Foc R1* and 105 SPs were identified from *Foc TR4* (Supplementary Table S1).

**Figure 2:**
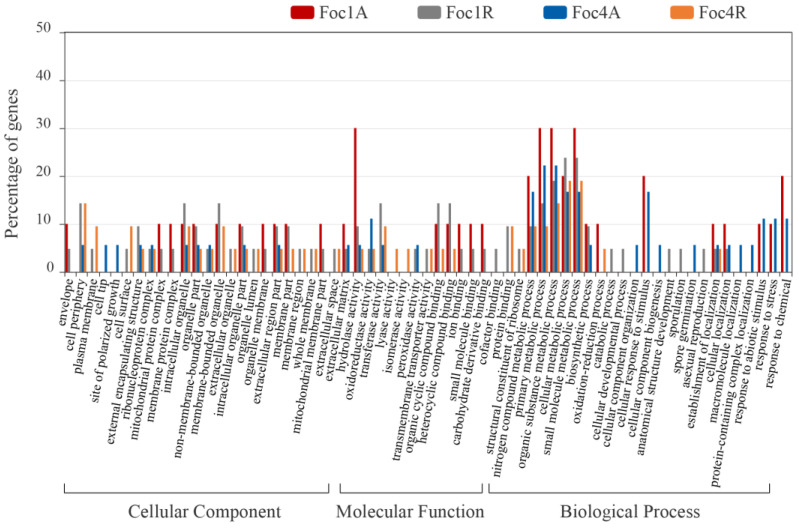
WeGO software (Version 2.0) analysis of SPs from *Foc R1* and *Foc TR4*. Foc1A and Foc4A represent SPs produced by *Foc R1* and *Foc TR4* culture alone, respectively; Foc1R and Foc4R represent SPs produced by *Foc R1* and *Foc TR4* culture with banana roots, respectively.

Through cellular component analysis, we found that these SPs were not only distributed in extracellular or surface regions, such as extracellular region part, extracellular matrix, external encapsulating structures, cell surface, and plasma membrane, but also distributed in intracellular regions, such as intracellular organelles, mitochondrial protein complex, and ribonucleoprotein complex (Figure 2). Our molecular function analysis showed that high proportion of SPs had catalytic activities of hydrolase activity, oxidoreductase activity, and transferase activity (Figure 2). In the biological process analysis, we found that these SPs were mainly concentrated in nitrogen compound metabolic process, primary metabolic process, organic substance metabolic process, cellular metabolic process, and small molecule metabolic process (Figure 2).

### 3.3. Analysis of Specific SPs of Foc R1 and Foc TR4 in Culture Alone and with Banana Roots

**Figure 3:**
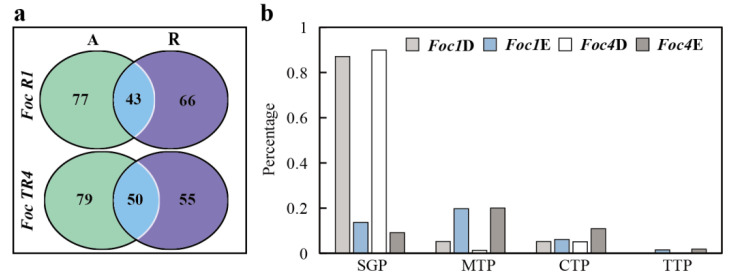
Analysis of specific secretory proteins (SPs) of *Foc R1* and *Foc TR4* in culture alone and with banana roots. (**a**) The comparation of SPs generated under culture alone and culture with banana roots. A, culture alone; R, culture with banana roots. Green circles indicate the SPs specially emerged under the condition of culture alone; purple circles indicate the SPs specially emerged under the condition of culture with banana roots. Two biological replicates were performed among the protein identified. (**b**) The ratio of SPs with different transit peptides. *Foc1*D, the SPs of *Foc R1* disappeared after culture with banana roots; *Foc1*E, the SPs of *Foc R1* appeared after culture with banana roots; *Foc4*D, the SPs of *Foc TR4* disappeared after culture with banana roots; *Foc4*E, the SPs of *Foc TR4* appeared after culture with banana roots. SGP, proteins with signal peptide; MTP, proteins with mitochondrial transit peptide; CTP, proteins with chloroplast transit peptide; TTP, proteins with thylakoid luminal transit peptide.

We observed that compared to *Foc R1* in culture alone, 66 new SPs emerged, and 77 SPs disappeared during culture with banana roots. After *Foc TR4* was cultured with banana roots, 55 new SPs emerged, and 79 SPs disappeared (Supplementary Table S2; Figure 3). The trends of SP emergence and disappearance shared by *Foc R1* and *Foc TR4* were basically the same, and some SPs with high abundance were also present in the emerging or disappearing groups (Supplementary Table S2).

Some SPs were undetectable during culture with banana roots, such as beta-cyclopiazonate dehydrogenase (BCD), D-galactonate dehydratase (DGD), 1,3-beta-glucanosyltransferase (Gel1), putative alpha-galactosidase D (AGD), catalytic protein (CP), lipase 1 (Lip1), 6-hydroxy-D-nicotine oxidase (6hDNO), putative ABC transporter anion-binding protein (ABCT), SSCRP protein (SSCRP), COPII coat assembly protein (sec16), and allergen Asp f4 ortholog (Aspf4o) (Supplementary Table S2). These SPs are multifunctional proteins and potential effectors. Evaluation of the gene expression levels of some of these SPs (Supplementary Figure S1) showed that most were not obviously changed or showed a moderate decrease after culture with banana roots, such as DGD and AGD. We speculated that the decrease in the expression of these SPs is largely due to protein accumulation or secretion, or possibly entry of high levels into the banana roots. However, although the *Foc* proteins in banana roots could be identified, it is hard to distinguish between SPs and cellular proteins.

Some SPs were identified only in *Foc R1* or *Foc TR4* in culture with banana roots, such as KatG, superoxide dismutase (SOD), putative 5-methyltetrahydropteroyltriglutamate (HcMt), malate dehydrogenase (MD), putative phosphoketolase (Pk), catalase-1 (Cat1), alanine/arginine aminopeptidase (AaAp), alcohol oxidase (AO), aconitate hydratase (AH), UPF0160 protein MYG1 (MYG1), putative peroxiredoxin pmp20 (pmp20), and S-formylglutathione hydrolase (SFgH) (Supplementary Table S2). These proteins included numerous protective enzymes, such as KatG, SOD, and Cat1, which might play roles in protecting *Foc R1* or *Foc TR4* against the defensive oxidative burst produced in infected plants. We also detected the gene expression levels of these SPs (Supplementary Figure S1). Interestingly, there was no clearly increase of any of these genes after culture with banana roots, and some genes, such as *Cat1* and *MYG1*, were even down regulated. The results further suggested that the newly emerged SPs were possibly regulated at the level of protein accumulation or secretion rather than at the transcriptional level.

Furthermore, we noted that the SPs that emerged after culture with banana roots were mostly unconventional SPs without signal peptide (57/66 for *Foc R1* and 50/55 for *Foc TR4*), while the SPs that disappeared after culture with banana roots were mostly conventional SPs with signal peptide (67/77 for *Foc R1* and 71/79 for *Foc TR4*) (Supplementary Table S2, Figure 3b). Further analysis of the SPs that were identified both in culture alone and in culture with banana roots revealed that there were no differences in the proportions of conventional and unconventional SPs (Supplementary Table S2). We also found that compared with conventional SPs, a large proportion of these unconventional SPs contained transit peptides, especially mitochondrial transit peptides (Figure 3b), indicating that these proteins might enter banana plant cells and some specific organelles to exert their corresponding functions.

### 3.4. Analysis of Foc R1- and Foc TR4-Specific SPs

**Figure 4:**
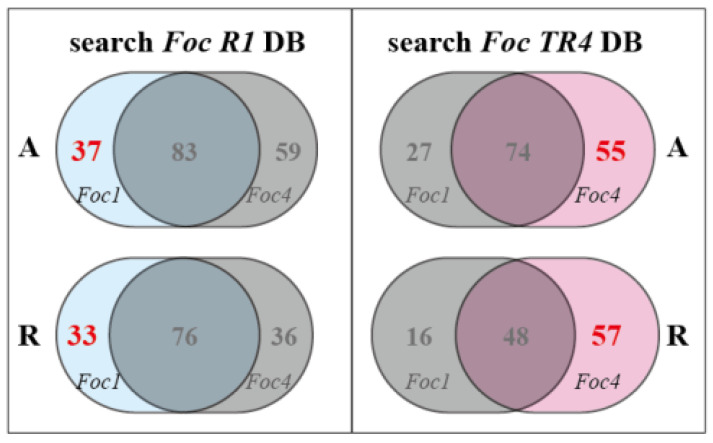
The comparation of SPs generated by *Foc R1* and *Foc TR4***.** Left, specific SPs of *Foc R1*, and the comparation was based on the search results against *Foc R1* database. Right, specific SPs of *Foc TR4*, and the comparations were based on the search results against *Foc TR4* database. A, culture alone; R, culture with banana roots.

**Figure 5:**
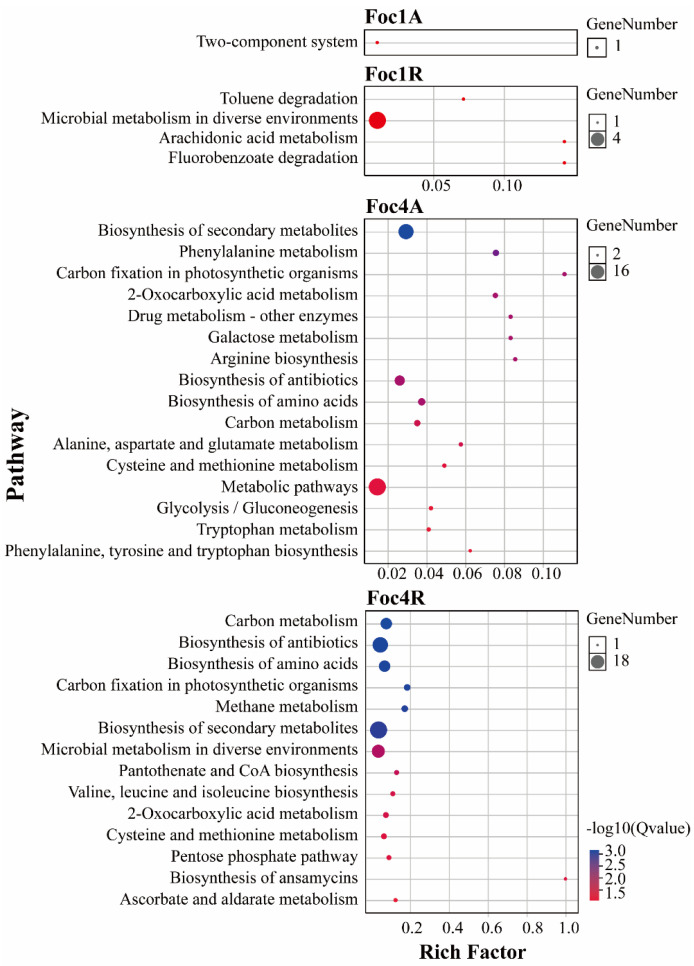
KEGG analysis of specific SPs of *Foc R1* and *Foc TR4*. Foc1A, the metabolic pathways participated by *Foc R1* specific SPs generated under culture alone; Foc1R, the metabolic pathways participated by *Foc R1* specific SPs generated under culture with banana roots; Foc4A, the metabolic pathways participated by *Foc TR4* specific SPs generated under culture alone; Foc4R, the metabolic pathways participated by *Foc TR4* specific SPs generated under culture with banana roots.

Based on the comparative analysis, we identified 37 *Foc R1*-specific SPs and 55 *Foc TR4*-specific SPs under conditions of culture alone, while and 33 *Foc R1*-specific SPs and 57 *Foc TR4*-specific SPs were identified under conditions of culture with banana roots (Supplementary Table S3; Figure 4). Only a few of *Foc R1*-specific SPs participated in the Kyoto Encyclopedia of Genes and Genomes (KEGG) metabolic pathways (MPs), with scores outside the confidence interval (Supplementary Table S4). A high proportion of *Foc TR4*-specific SPs were involved in various MPs. Eighteen *Foc TR4*-specific SPs identified under conditions of culture alone were involved in 30 different MPs, of which, 10 had scores within the confidence interval. Thirty-one *Foc TR4*-specific SPs identified under conditions of culture with banana roots were involved in 55 MPs, of which, eight had scores within the confidence interval. *Foc TR4*-specific MPs included carbon metabolism, biosynthesis of secondary metabolites, carbon fixation in photosynthetic organisms, amino acid biosynthesis, antibiotic biosynthesis, phenylalanine metabolism, and cysteine and methionine metabolism (Supplementary Table S4; Figure 5). These variations may be related to the different abilities of the two races to infect Cavendish banana plants. We found that some *Foc TR4*-specific SPs were involved in multiple MPs, such as catalase-peroxidase 2 (KATG2s), which is involved in both phenylalanine metabolism and drug metabolism pathways, and O-acetyl homoserine (thiol)-lyase (OASTL), which is involved in antibiotic biosynthesis, cysteine and methionine metabolism pathways, sulfur metabolism, and four other MPs (Supplementary Table S4).

We also analyzed the expression of some specific SPs at the transcriptional level, including *pectin esterase* (*PE*), *secreted in xylem 1* (*Six1*), *secreted in xylem 6* (*Six6*), *stabilin-2* (*Sta-2*), *lipase* (*lipP*), *lysophospholipase* (*LPL*), *thioredoxin* (*TD*), *bikaverin cluster-transcription factor enhancer* (*BctfE*), *plant elicit peptide 1* (*PEP1*), and *surface protein 1* (*SP1*) in *Foc R1*, and *KatG2*, *DJ-1 family protein* (*DJ-1*), *choline dehydrogenase* (*ChD*), *serine-rich protein* (*SRP*), *L-sorbosone dehydrogenase* (*LSD*), *invertase 2* (*Inv2*), *OASTL*, *trehalase* (*Treh*), *necrosis and ethylene-inducing like protein* (*NLP*), and *N1-acetylpolyamine oxidase* (*N1ApO*) in *Foc TR4* (Supplementary Figure S2). The results showed that the gene expression of the majority of *Foc R1*- and *Foc TR4*-specific SPs showed similar trends at both transcriptional and secretory levels, with a few exceptions, such as *PE* and *LSD*, which showed opposite patterns of change.

### 3.5. Knockout of Root-Induced Foc TR4-Specific OASTL Resulted in the Loss of Pathogenicity of Foc TR4 in Banana

**Figure 6:**
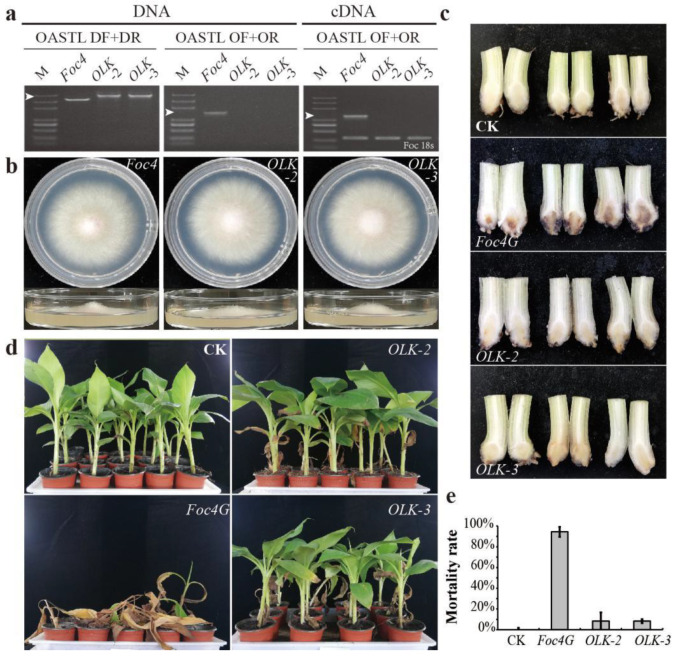
Effect of OASTL knockout on *Foc TR4* pathogenicity to banana. (**a**) Molecular identification of OASTL knockout strains of *Foc TR4* (*OLK*); (**b**) morphological comparison of *Foc4* and *OLK* strains after inoculation on PDA medium for 5 days; (**c**) observation of the pseudo-stem section of bananas 3 days after inoculation with *Foc4G* or *OLK*; (**d**) observation of the plant statue of bananas 30 days after inoculation with *Foc4G* or *OLK*; (**e**) the survival rate of banana plants inoculated with *Foc4G* or *OLK*. M, DL5000 marker; DF+DR, amplification with primers outside the homologous recombination arms of *OLK*; OF+OR, amplification of open reading frames of *OLK*; the white arrows in an indicate the location of the objective bands; *Foc4G*, GFP fluorescent strain of *Foc TR4*; three biological replicates were performed.

Based on our analysis of the identified SPs of *Foc R1* and *Foc TR4*, we found that OASTL was one of the few root-induced and *Foc TR4*-specific SPs present at high abundance (Table 1). OASTL does not have signal peptide, nor some other predictable transit peptide (Supplementary Table S2). The gene expression level of *OASTL* in *Foc TR4* was much higher than that in *Foc R1*, and *OASTL* was one of the few genes that had a higher expression level after culture with banana roots (Supplementary Figure S2). OASTL encodes a key enzyme in the biosynthesis of cysteine, which is the precursor of an enormous number of sulfur-containing defense compounds, such as glutathione (GSH) and methionine. Therefore, we conducted further research focusing on OASTL. Through homologous recombination, we obtained the OASTL knockout (*OLK*) strains of *Foc TR4* (Figure 6a). *OLK* strains grew well on the normal PDA medium (Figure 6b). *Foc TR4* control strain (*Foc4G*) and *OLK* strains were then used to infect banana ‘Brazil’ and after 3 days, the pseudo-stem section of bananas infected by *Foc4G* had turned black, whereas no obvious blackening was observed in the pseudo-stems infected by *OLK* (Figure 6c). One month after the infection, almost all the bananas infected by *Foc4G* had died, while 91.7% of those infected by *OLK* survived (Figure 6d,e). These results suggested that OASTL represented a key difference between *Foc R1* and *Foc TR4*, which seriously affects the pathogenicity of *Foc TR4* in Cavendish banana plants.

## 4. Discussion

In this study, we identified the SPs produced by *Foc R1* and *Foc TR4*, and expected to find out some relationships between the SPs and the pathogenicity of *Foc TR4*. Pathogens secrete SPs into the cytoplasm or apoplast to interact with the host. Cell wall degrading enzymes (CWDEs) are one of the major types of SPs [23]. In microorganisms, the ability to degrade plant cell walls is widespread, a function that is mediated mainly by enzymes including pectinase, hemicellulase, and cellulase [23]. Pectin esterase (PE), endoglucanase (BG), arabinase, and a group of glycosidases, such as galactosidase, glucosidase, and arabinofuranosidase were identified among the SPs of both *Foc R1* and *Foc TR4* (Supplementary Table S1). In addition, these enzymes could be related to plant cell wall degradation, some enzymes may be related to fungal cell wall modification, such as chitinase, glucan 1,3-beta-glucosidase, glucan 1,6-beta-glucosidase, and 1,3-beta-glucanosyl-transferase, were also identified among the SPs of both *Foc1* and *Foc4* (Supplementary Table S1). Chitin and β-glucan, both of which are fungal cell wall polysaccharides, also serve as pathogen-associated molecular patterns (PAMPs) in plant. Pathogen-secreted chitinases with only binding ability prevent chitin-triggered immunity by sequestering chitin fragments [24], while the roles of 1,3-beta-glucosidases secreted by plant pathogens are largely unknown [25]. The functions of glucan 1,3-beta-glucosidases are well characterized in mammalian pathogens, allowing fungi to avoid host immune stimulation by β-glucan through secretion of endo-1,3-beta-glucosidase to remove any exposed β-glucan polysaccharide [26]. Whether the same situation exists in *Foc R1* and *Foc TR4* and other plant pathogens remains to be verified. On the other hand, the callose deposited by plants in response to pathogen invasion is also a type of glucan endo-1,3-beta-glucon [27]. Beta-1,3-glucanase expression by plants reduced callose deposition and facilitated invasion by pathogens [28,29]. Therefore, it can be speculated that the secretion of beta-1,3-glucanase and beta-1,6-glucanase by *Foc R1* and *Foc TR4* represents a mechanism by which pathogens eliminate callose and promote invasion. The 1,3-beta-glucanosyltransferases also play a significant role in structural modification of the fungal cell wall during plant infection [30]. In addition to the enzymes previously mentioned, some other hydrolases, such as extracellular protease, endopeptidase, and ribonuclease, were also identified among the SPs shared by *Foc R1* and *Foc TR4* (Supplementary Table S1).

Protein secretion by fungi can occur either via the ER-Golgi-vesicle-dependent route (conventional protein secretion, CPS) or alternative routes classified as unconventional protein secretion (UPS) [31]. While the CPS machinery is well documented in fungi, the UPS machinery has not been well elucidated [31]. From the SPs generated in *Foc R1* and *Foc TR4* in culture with banana roots, we found that unconventional SPs without signal peptide were significantly increased (Supplementary Table S2; Figure 3b). In the study of *F. graminearum*, it was also found that a relative lower ratio of the extracellular proteins contained signal peptide during wheat heads infection, which was considered to be partly due to the fungal lysis occurred during pathogenesis [32]. We speculated that the reasons for significantly increase of SPs without signal peptide in this study could be caused by both UPS and fungal lysis, but it needs to be determined which was the main factor. We have previously identified mycelium proteins of *Foc R1* and *Foc TR4* from two dimensional polyacrylamide gel electrophoresis (2-DE) gels [32]. The proteins identified from 2-DE gels are usually relatively high abundant proteins of the organism. However, only a few of these mycelial proteins [33] appeared in the SPs that newly emerged after co-culture with banana roots, similar to the amount they appeared in the SPs that disappeared after co-culture with banana roots (Supplementary Table S2). The result suggested that the emergence of these unconventional SPs might be attributed to UPS to a greater extent. This holistic change suggests that it might not to be generated by the direct control of multiple SPs acting individually, but by the overall control of certain secretion systems. Fungi seem to have the ability to control the whole secretory system [34], which might be more convenient for rapid and efficient switching of SPs in response to different environments, and could also be one of the reasons for the existence of these different types of secretory systems. In the early stages of the invasion process, *Foc R1* and *Foc TR4* clearly use more SPs associated with the UPS routes than the CPS routes (Figure 3b).

Among the SPs associated with the UPS route produced by *Foc R1* and *Foc TR4* cultured with banana roots, enzymes of the reactive oxygen species (ROS) scavenging system were one of most recognizable groups. The SPs of *Foc R1* and *Foc TR4* that emerged during culture with banana roots both contained various antioxidative enzymes, including CAT, KatG, and SODs (Supplementary Table S2). It is well documented that plant cells produce ROS as one of the earliest cellular responses (oxidative burst) to pathogen attack [35]. This suggests that *Foc R1* and *Foc TR4* respond to oxidative challenges mounted in banana plants by activating the accumulation or secretion of these antioxidative enzymes. However, we also found that the expression of two KatG2s disappeared or was greatly reduced during culture with banana roots (Supplementary Table S2). Phylogenetic analyses showed that most phytopathogenic fungi have an intracellular KatG protein (KatG1), and an extracellular KatG2 with an N-terminal signal sequence for secretion [36]. Studies of KatGs in the maize pathogen *Fusarium verticillioides* suggested that KatG1 and KatG2 have the function of H_2_O_2_ degradation intracellularly and extracellularly, with KatG1 conferring greater H_2_O_2_ tolerance than KatG2 [36]. In this study, we found that KatG1 was also exist extracellularly (Supplementary Table S2).

In this study, we identified some reported plant pathogen effector proteins, such as chorismate mutase, which blocks salicylic acid synthesis [9,37], as well as some possible effector proteins, such as Antigen1 and Enolase-like proteins (Supplementary Table S1). Antigen1, which is an allergen protein originally identified from *Aspergillus fumigatus*, recruits human immune regulators of immune evasion and cell damage [38]. Enolase is one of the key enzymes in glycolysis, and has also been reported in an animal pathogen that requires the combination of enolase and β-1,3-glucanase for the complete virulence [39]. On the other hand, some of the previously reported effectors of *Foc TR4*, including FocCP1 [17,20], Six1a [18], and Six8 [19] have not been identified (Supplementary Table S1). In two recent articles describing the function of FocCP1 in banana infection, one reported that this protein was required for full virulence of *Foc TR4* [20], while the other considered that it triggered immune reaction [17]. However, in this study, FocCP1 was not identified in *Foc TR4* (EMT67648.1), and only a small number of the matched peptides were identified in *Foc R1* (ENH63703.1) (Supplementary Table S1). Secreted in xylem (Six) proteins, which are the most well-known effectors of *F. oxysporum*, were originally identified in *F. oxysporum* f. sp. *lycopersici* [40,41], and many members have been predicted from the genome of *Foc R1* and *Foc TR4* [42]. However, in this study, only Six1, Six6, and Six9 were identified among the SPs of *Foc R1* or *Foc TR4*. The Six1 homologue (Six1d) was identified in *Foc R1*, while the Six1 homologues proteins (Six1a, Six1b, Six1c) were not identified in *Foc TR4* (Supplementary Table S1). This finding seems to be inconsistent with the absolute requirement for Six1a for full toxicity of *Foc TR4* [18], while it is also possible that Six1s does not play a role in the early stage of invasion by *Foc TR4*.

The SPs of *Foc TR4* identified in this study were involved in various metabolic pathways, and might contain some potential effectors (Figure 5). We found that most of the SPs meeting the criteria of being root inductive (Supplementary Table S2) and *Foc TR4*-specific were present at relatively low abundances, with OASTL being one of the few SPs present at relative high abundance (Table 1; Supplementary Table S3). OASTL is a key enzyme in the biosynthesis of cysteine, which is a reduced sulfur donor molecule occupying a central position in plant primary and secondary metabolism [43]. As such, OASTL is widely involved in the MPs of carbon metabolism, amino acid biosynthesis, and antibiotic biosynthesis (Supplementary Table S4). Cysteine plays an important role in plant immunity through its involvement in the synthesis of many defense compounds, including the antioxidant GSH [44], phytoalexins [45], and the precursor of the stress response regulating hormone, ethylene [46]. The cytosolic cysteine content in the *Arabidopsis* OASTL mutant *oas-a1* is decreased, and the mutant plants suffer from oxidative stress due to ROS accumulation [47]. The *oas-a1* plants lack effector-stimulated immune hypersensitivity (HR), and are more sensitive to both of the hemibiotroph and necrotroph pathogens [48,49]. Knockout of the *OASTL* gene did not affect the growth and development of *Foc4* on PDA medium (Figure 6b), but seriously damaged the pathogenicity of *Foc TR4* in banana ‘Brazil’ (Figure 6c,d), which suggested that OASTL could be an important effector of *Foc TR4*. As an effector protein of *Foc TR4*, the reason for the existence of OASTL is certainly not to improve the immune capacity of plants as mentioned previously, and we speculated that it might interfere with cysteine metabolism in banana via a mechanism that is yet to be clarified.

In this study, we extracted and identified SPs from *Foc R1* and *Foc TR4*, and found that there were a large number of hydrolases, oxidoreductases, transferases, and binding proteins among the SPs shared by *Foc R1* and *Foc TR4*. During culture with banana roots, *Foc R1* and *Foc TR4* both secreted many unconventional SPs, suggesting that *Foc R1* and *Foc TR4* strengthen the unconventional secretion pathway under banana root induction. We also found that *Foc TR4* generated more specific SPs and had a higher proportion of SPs involved in various metabolic pathways than *Foc R1*. Among these root inductive *Foc TR4*-specific SPs, our results implicated the cysteine biosynthesis enzyme-like protein OASTL as an important effector of *Foc TR4*. Overall, our results provide a useful foundation for understanding the pathogenic mechanisms underlying banana-*Foc TR4* interactions.

## Figures and Tables

**Table 1:** The Root Inductive and *Foc TR4* Specific SPs.

Accession	Num. of Significant Matches								Description
	F1GA-1	F1GA-2	F1GR-1	F1GR-2	F4GA-1	F4GA-2	F4GR-1	F4GR-2	
EMT72692.1	11				8		72	21	O-acetylhomoserine (thiol)-lyase
EMT64128.1			9		11		40	6	S-formylglutathione hydrolase
EMT70691.1			19		17		20	8	Malate dehydrogenase, mitochondrial
EMT64225.1	3	8	3		8		31	4	Putative beta-glucosidase A
EMT66678.1	19		11		12		19	7	SDO1-like protein C21C3.19
EMT68479.1			13				33	2	Phosphoserine aminotransferase
EMT70909.1			15				27	5	Dihydroxy-acid dehydratase
EMT63756.1			2				27	4	Cys-Gly metallodipeptidase dug1
EMT68383.1			11		5		19	7	hypothetical protein FOC4_g10012800
EMT66833.1			10		4		17	8	Cytochrome c peroxidase, mitochondrial
EMT64384.1							26	2	hypothetical protein FOC4_g10010883
EMT68365.1	4		21		5		17	6	hypothetical protein FOC4_g10012782
EMT60820.1	8		10			5	14	5	Putative branched-chain-amino-acid aminotransferase TOXF
EMT72882.1			9			2	18	4	Putative alpha-N-arabinofuranosidase C
EMT73358.1			10				12	12	Ubiquitin-40S ribosomal protein S27a
EMT68567.1			16				15	8	Serine hydroxymethyltransferase, cytosolic
EMT70711.1			16				19	3	Putative Xaa-Pro aminopeptidase P
EMT60464.1					4		9	8	hypothetical protein FOC4_g10011735
EMT62355.1		5	3			7	9	4	Histone H4
EMT69138.1			7				14	6	Cytochrome P450 55A1
EMT61471.1							5	12	Aldehyde dehydrogenase
EMT63560.1					7		6	4	Transketolase
EMT69534.1	3		6		3		7	7	hypothetical protein FOC4_g10003264
EMT73283.1	4	2	3		9		6	2	Cytochrome c
EMT61353.1			17				13	2	hypothetical protein FOC4_g10014408
EMT68171.1			3		5		4	6	Nucleoside diphosphate kinase
EMT68727.1							13	2	hypothetical protein FOC4_g10000306
EMT65157.1							9	4	60S ribosomal protein L4-B
EMT60471.1			9				10	2	Glutathione S-transferase omega-like 2
EMT66944.1					2		2	4	Aldose reductase A
EMT73111.1	4		6				5	3	Coproporphyrinogen-III oxidase
EMT62349.1					2		3	2	14-3-3 protein like protein
EMT66456.1			3				2	4	Inositol monophosphatase 2
EMT70972.1							4	2	Fructose-bisphosphate aldolase
EMT66185.1							2	3	Putative oxidoreductase C26H5.09c
EMT65501.1							2	2	40S ribosomal protein S18

Note: Accession, the protein accession number in the NCBInr database; Num. of significant matches, the number of matched peptides in LC-MS identification; F1GA-1, F1GA-2, the two biological replicates of identified SPs generated by *Foc R1* under culture alone; F1GR-1, F1GR-2, the two biological replicates of identified SPs generated by *Foc R1* under culture with banana roots; F4GA-1, F4GA-2, the two biological replicates of identified SPs generated by *Foc TR4* under culture alone; F4GR-F4-1, F4GR-F4-2, the two biological replicates of identified SPs generated by *Foc TR4* under culture with banana roots; Description, descriptions of the target proteins that were obtained from the annotations of the protein database of *Foc TR4* (BioProject: PRJNA174275, 14,459 component sequences deposited in the NCBInr).

## Supplementary Materials

The following are available online at https://www.mdpi.com/2218-273X/10/10/1430/s1, Table S1: The SPs generated by *Foc R1* and *Foc TR4* under culture alone or culture with banana roots; Table S2: The *Foc* SPs that disappeared or emerged after culture with banana roots; Table S3: The specific SPs of *Foc R1* and *Foc TR4*; Table S4: The KEGG pathways that specific *Foc* SPs involved; Table S5: The primers used for RT-PCR analysis and vector construction. Supplementary Figure S1: RT-PCR analysis of the genes corresponding to the SPs disappeared or emerged after culture with banana roots; Supplementary Figure S2: RT-PCR analysis of the genes corresponding to specific SPs of *Foc R1* or Foc *TR4*; Supplementary Figure S3: pCT74HP-eGFP.
